# Vision-Related Quality of Life in Danish Patients with Albinism and the Impact of an Updated Optical Rehabilitation

**DOI:** 10.3390/jcm12175451

**Published:** 2023-08-22

**Authors:** Kristian Lisbjerg, Joaquim Torner Jordana, Vibeke N. Brandt, Christine Kjølholm, Line Kessel

**Affiliations:** 1 Department of Ophthalmology, Copenhagen University Hospital—Righospitalet, 2600 Glostrup, Denmark; kristian.lisbjerg@regionh.dk (K.L.); joaquim.torner.jordana@regionh.dk (J.T.J.); vibeke.neiiendam.brandt@regionh.dk (V.N.B.); christine.kjoelholm@regionh.dk (C.K.); 2 Department of Clinical Medicine, University of Copenhagen, 2200 Copenhagen N, Denmark

**Keywords:** albinism, optical rehabilitation, quality of life

## Abstract

(1) Background: Albinism is characterized by a lack of pigment in eyes, hair, and skin and developmental changes in the eye such as foveal hypoplasia. Patients require optical rehabilitation due to low vision, refractive errors, and photosensitivity. We aimed to assess vision-related quality of life in patients with albinism and to evaluate how this was affected by optical rehabilitation. (2) Methods: Patients with ocular or oculocutaneous albinism were invited for the study. Free-of-charge optical rehabilitation was provided as needed, including filters, glasses for near or distance, contact lenses, magnifiers or binoculars. Vision-related quality of life was assessed prior to and after optical rehabilitation using the visual function questionnaire (VFQ39) and the effect of optical rehabilitation was evaluated after accounting for age, gender, and visual acuity. (3) Results: Seventy-eight patients filled out the VFQ39 at the initial visit. Fifty patients (64.1%) returned the questionnaire 3–6 months after optical rehabilitation. The mean age of included patients was 35.9 years (standard deviation 16.6), and their best corrected distance visual acuity was 56 ETDRS letters (range 3–81). The VFQ39 composite score improved significantly from a median of 62.5 (range 14.2–77.0) to 76.5 (20.6–99.6). Significant improvements were seen for ocular pain, social functioning, mental health, role difficulties, and dependency, whereas self-assessed distance or near visual functions did not change. (4) Conclusions: Optical rehabilitation improved the self-reported vision-related quality of life in Danish patients with albinism on a number of parameters related to leading an independent and worry-free life, whereas visual improvement for distance and near tasks was likely limited by the nature of the disease and by the fact that most patients already had access to some optical aids prior to the study.

## 1. Introduction

Albinism is a rare condition characterized by decreased pigmentation in skin, hair, and eyes [1]. The prevalence of oculocutaneous albinism in European populations is estimated at 1 in 10,000 to 1 in 15,000 [2], whereas prevalence data for ocular albinism are not available. Albinism is caused by variants in genes associated with melanocyte differentiation, melanin synthesis, and melanosomal proteins [3]. The lack of pigment increases the risk of ultraviolet-induced skin changes, including cancers, which can be found in high numbers of patients with albinism living in Africa [4]. Patients with albinism often stand out due to their fair appearance. In certain parts of the world, people with albinism may be excluded or persecuted by their communities and they may fall victim to witchcraft-related violence [4].

In addition to the lack of pigmentation, patients with albinism have low vision due to maldevelopment of the fovea [5] and abnormal decussation at the optic chiasm, with the majority of optic nerve fibers crossing to the contralateral cerebral hemisphere [6] and nystagmus. Albinism is a common cause of visual impairment in childhood [7,8].

Visual impairment may pose an obstacle to participating in educational and leisure time activities which may further contribute to the stigma and rejection from society experienced by many people with albinism [9]. All these factors may influence self-perceived quality of life, and especially vision-related quality of life. There is little knowledge on the quality of life of patients with albinism living outside Africa. A Brazilian study showed that patients with albinism rated their physical quality of life lower than a control group [10]. An American study found a high prevalence of psychiatric comorbidity and a low self-rated, vision-specific quality of life, especially for mental health and social functioning [11].

We conducted the present study on self-reported, vision-related quality of life using the 39-item visual function questionnaire (VFQ39) in a cohort of Danish patients with albinism. The study was part of a larger study aimed at describing genotype–phenotype associations and establishing evidence for optical rehabilitation in patients with albinism. Results of this part of the study have been published previously [12,13]. Furthermore, we evaluated whether the vision-related quality of life changed after an updated optical rehabilitation.

## 2. Materials and Methods

Patients who had previously been seen at the national Eye Clinic at the Kennedy Center with a clinical diagnosis of ocular or oculo-cutaneous albinism were invited for an examination including evaluation by an ophthalmologist and low vision optometrist. The Eye Clinic is a tertiary referral center specializing in rehabilitation and follow-up of patients with low vision mainly due to genetic diseases. Thus, all participants had at some point earlier in their life received optical rehabilitation. The genotype and phenotype of patients in the study have previously been reported [12].

All included patients underwent a detailed examination that included determination of best corrected distance vision using retroilluminated ETDRS charts (Precision Vision, Woodstock, IL, USA). In short, different charts were used for each eye and for the binocular measurements. Visual acuity was calculated as the number of letters read correctly at 4 m distance plus 30. If fewer than 20 letters were read correctly at 4 m distance, the chart was moved to 1 m distance and visual acuity was calculated as the number of letters read correctly on the first 6 lines at 1 m plus the number of letters read at 4 m distance. This results in a maximum score of 100. Near visual acuity was measured using the Colenbrander mixed contrast card (Precision Vision) at the patient’s preferred distance with appropriate correction for near distance; the near visual acuity was corrected for reading distance. Contrast vision was measured using the Pelli–Robson chart at 1 m under standard illumination. An addition of +0.75 Diopter were added for testing at 1 m distance. Subjective and objective refractioning was performed by a low vision optometrist. Objective refractioning was obtained using retinoscopy and autorefraction using a Retinomax (Righton Retinomax, K-plus 3, Tokyo, Japan). Ophthalmological examination included slit lamp examination visualizing the anterior and posterior segments of the eye. Photographic images were captured of the iris and fundus and were used to grade the level of iris transillumination and fundus pigmentation as described using the methods described by Wang et al. for iris transillumination [14] and Kruijt et al. for fundus pigmentation [15]. The degree of foveal development was evaluated using the grading system developed by Thomas et al. [16] based on 3D optical coherence tomography scans covering 6.0 × 6.0 mm, 512 × 128 using a Topcon OCT-2000 (Topcon cooperation, Tokyo, Japan).

As part of the examination, patients were offered optical rehabilitation with glasses or contact lenses, including filters and low vision optical aids as needed [13]. Patients were instructed in the use of the optical aids before administration. Optical rehabilitation was offered free of charge to participants, as optical aids for disease-related photosensitivity are reimbursed by the local health care system. Optical rehabilitation included glasses for distance and near work, light attenuation by filters, and loupes or binoculars according to the participant’s need. Participants were presented with a broad range of colored and neutral filters and were given the opportunity to test the filters indoors and outdoors and for near and distance tasks before deciding on a filter of choice. First, participants were instructed to determine the desired degree of light attenuation using a neutral (grey) filter before they compared to colored filters. A full list of filters included in the study have been published previously [13].

On the day of the examination, the visual function questionnaire 39 (VFQ39) was administered in a Danish version [17] on a tablet to allow the participants to magnify the text or set illumination levels as preferred. The VFQ39 was developed by the National Eye Institute and can be used to evaluate the vision-related quality of life in people with low vision. The questionnaire includes questions on general health and vision. The items in the questionnaire can be subdivided into 12 categories: general health, general vision, ocular pain, near and distance activities, color and peripheral vision, vision-specific social functioning, mental health, role difficulties, dependency, and driving. Each subscale contains a number of questions and the subject is asked to rate their response on an ordinal scale. A score is calculated for each subscale and finally a composite score can be calculated. The score ranges from 0 (worst possible score) to 100 (best possible score). The same questionnaire was emailed or mailed in a print version to participants 3–6 months after the visit to the Kennedy Center depending on the preferred mode of contact indicated by participants at the visit. One reminder (e-mail or mail version) was sent in case of no response.

Statistical analyses were performed using SigmaPlot (SigmaPlot for Windows Version 13.0, Systat Software, Inc., Düsseldorf, Germany). The VFQ39 was evaluated and analyzed according to the recommendations by the National Eye Institute [18]. Relationships between best corrected visual acuity (distance and near) and VFQ subscale scores were evaluated with multiple regression models adjusted for age and gender. The paired VFQ observations were compared with a paired *t*-test or Wilcoxon signed rank test depending on the distribution of data. Results were controlled for age as a confounding variable with analysis of covariance (ANCOVA). A *p*-value of 0.05 was considered significant.

## 3. Results

A total of 120 patients with albinism were invited to participate in the study; 92 agreed to participate. The visual function questionnaire (VFQ39) was administered to patients aged 15 years or older (*n* = 85); 78 patients filled out the questionnaire prior to an updated optical rehabilitation and 50 participants (64.1%) returned the questionnaire 3 to 6 months after optical rehabilitation. Very few patients had a visual acuity that allowed them to hold a driver’s license (*n* = 12) and fewer (*n* = 8) had a driver’s license. For this reason, questions regarding driving were omitted from the results below. The mean age of included participants was 35.9 years (standard deviation 16.6), and 43 (55%) were male. The median best corrected distance visual acuity was 56 ETDRS letters (range 3–81) and the median contrast sensitivity was 1.95 (range 0.6–2.1). Baseline characteristics of included participants are summarized in Table 1.

Two patients did not use any optical aids before the study and one continued not to use optical aids after the study. The patients used many different combinations of optical aids before the study, with a median of three optical aids per patient (range 0–8). Most commonly, patients would have a combination of glasses with and without filters (*n* = 42 patients), either alone (*n* = 16) or in combination with loupes (*n* = 20), in combination with binoculars (*n* = 7), or in combination with loupes and binoculars (*n* = 8). Four patients had glasses without filters or any other optical aids prior to the study and 15 had glasses with filters without any other optical aids prior to the study. As part of the study, patients were offered an updated optical rehabilitation according to their needs. A total of 144 optical aids were prescribed including glasses with filters (*n* = 64 patients received new glasses; eight were given to patients who did not have filter glasses before), glasses without filters (*n* = 25 patients were given new glasses; six were given to patients who did not have glasses without filters before), binoculars (*n* = 25; 17 were given to patients who did not use binoculars before), loupes (*n* = 18; six were given to patients who did not use a loupe before), and contact lenses (*n* = 3). Most patients received just one new pair of glasses with, or a new pair of glasses without filters, but some patients received several pairs, with a maximum of three new sets of filter glasses to accommodate for use under different light levels and viewing distances. In addition, most patients received just one set of loupes or binoculars but some received two, e.g., both a handheld and a head-mounted loupe, or both a set of binoculars to be used by one hand and a set to be used by both hands. Thus, the median number of optical aids that each patient would have after the study was five (range 0–12).

The VFQ39 questionnaire was administered to patients before and after the updated optical rehabilitation. The main results of the questionnaire were divided into the subscales (general health, general vision, ocular pain, near activities, distance activities and vision-specific mental health, role difficulties, dependency, color vision, and peripheral vision) and are summarized in Table 2.

**Figure 1:**
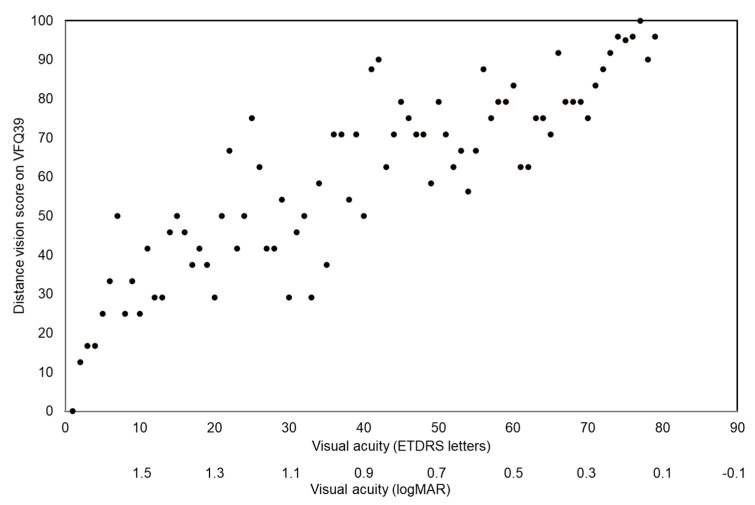
Association between distance visual acuity measured on the ETDRS chart and self-rated distance visual function before optical rehabilitation.

There was a high degree of correlation (r = 0.45) between best corrected distance visual acuity and distance vision activity function on the VFQ39 questionnaire, see Figure 1. There was no correlation between contrast vision measured on the Pelli–Robson chart and VFQ39 composite score or general vision (r = 0.38 and r = 0.24, respectively).

When adjusted for age and gender, best corrected distance visual acuity showed a significant positive relation to the VFQ39 subscales “general vision” (*p* = 0.005), “distance activities” (*p* < 0.001), “social functioning” (*p* < 0.001), and “dependency” (*p* = 0.03) but not to “mental health” (*p* = 0.1) or “role difficulties” (*p* = 0.8). Distance visual acuity also had a significant and better association with the “near activity” subscale (*p* = 0.001) than near visual acuity (*p* = 0.4). “Distance activities” was the only subscale significantly influenced by age (*p* = 0.05) and gender (*p* = 0.02), where a young female individual would score less on their self-reported distance activity vision than an older individual if their BCVA was the same.

Prior to optical rehabilitation, the lowest scores were given for “role difficulties”, “dependency”, and “mental health” followed in ascending order by “general vision”, “distance activities”, “near activities”, “general health”, “ocular pain”, and “social functioning”. The “color vision” and “peripheral vision” subscales received the highest scores. The maximum possible score was given by a substantial number of participants for “color vision”, “peripheral vision”, and “social functioning”.

**Figure 2:**
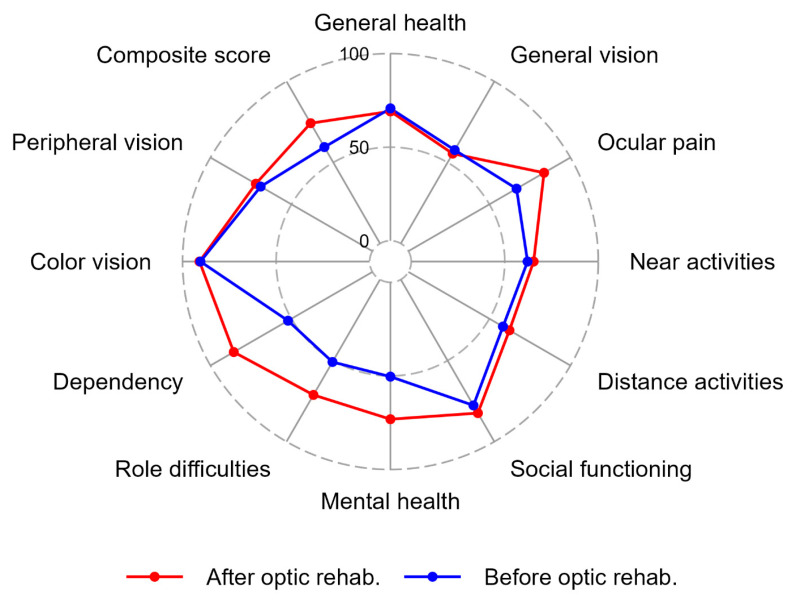
The radar plot demonstrates the median values of the 50 participants who filled out the VFQ39 questionnaire before and after the updated optical rehabilitation. The plot shows the eleven VFQ39 subscales and the composite score before and after optical rehabilitation. The lowest possible score was 0 and the maximum score was 100.

A number of items improved significantly after the updated optical rehabilitation, see Figure 2. These items included the composite score, “ocular pain”, “social functioning”, “mental health”, “role difficulties”, and “dependency”. Other items were unaffected by optical rehabilitation. These items included “general vision”, “general health”, “color vision”, “near activities”, “distance activities”, “role difficulties”, and “peripheral vision”. We controlled for age as a confounding variable to the paired observation differences, and only in “social functioning” did age act as a confounder in the analyses (*p* = 0.05).

## 4. Discussion

We evaluated the vision-related quality of life in Danish patients with ocular or oculo-cutaneous albinism using the VFQ39 questionnaire. The composite score was relatively low prior to optical rehabilitation (median score was 62.5) compared to patients with advanced glaucoma (>80 [20]), cataract (>70 [21]) or autosomal dominant optic atrophy (~70 [22]). The scores improved after an updated optical rehabilitation. The “color vision” and “peripheral vision” subscales were rated high by participants at both time points. These items are not expected to be affected by albinism although no specific tests for color vision or visual fields were performed as part of this study.

Typically, optical rehabilitation aims at improving distance and near function by enlarging objects, e.g., binoculars for distance viewing, or magnifiers for short distance viewing, or improving contrast function, or reducing the amount of discomfort from light by filters. Surprisingly, participants did not report improvements in distance or near vision after optical rehabilitation. All participants had been seen at some point prior to this study at our department and had thus received optical aids as needed at some point in their life, but many received new optical aids as part of the study. Most commonly, the aids given to participants included indoor and outdoor spectacles, and most often the new set of spectacles included a filter. Details of the optical rehabilitation have been published previously [13]. One reason that participants did not report improvements in distance and near vision could be that they all had some sort of optical aid to assist with near and distance tasks prior to the study; another reason is that their potential for visual improvement is limited due to the nature of the disease with maldevelopment of the fovea.

Photosensitivity is a common complaint in patients with albinism due to the lack of pigment in the iris and retina [13]. The term “photosensitivity” comprises all the problems patients may have with light entering their eyes, from visual disability to discomfort and pain. Tinted contact lenses, or spectacles, are useful in patients with albinism to alleviate the problems with photosensitivity [23]. Photosensitivity is not directly assessed by the VFQ39, but it includes questions on ocular pain. Participants reported significantly less ocular pain after the updated optical rehabilitation, and notably 44% reported the best possible score for ocular pain after rehabilitation compared to 1.3% prior to rehabilitation. We ascribe this result to the large number of, primarily, spectacles with filters [13] that were prescribed as a consequence of the study. In addition to filter glasses (or contact lenses) some patients may have chosen to alleviate photophobia by using caps or sunshields but the latter were not provided as part of the study.

The largest numerical improvements were seen for dependency and mental health. The questions related to dependency included whether participants preferred to stay at home, or did not leave the home without assistance, or if they depended on others’ opinions or help/assistance. Questions related to mental health included worries about one’s own vision or embarrassing others because of their vision, feeling frustrated or annoyed about their vision, or less in control of daily actions due to their vision. It is not clear if the improvements were related to the effect of new optical aids or whether participating in a study and learning more about the disease was the driving force behind the improvement, as interaction with healthcare providers or getting to know more about one’s disease may improve well-being. Optical rehabilitation has been shown to improve vision-related quality of life in patients with age-related macular degeneration, especially for role difficulties and dependency [24]. Mental health problems are common in patients with visual impairment [25]. Alleviating the mental consequences of visual impairment may prove to be of tremendous importance to patients and as such, it seems that the optical rehabilitation provided in our study was beneficial.

The effect of optical rehabilitation on vision-related quality of life has not previously been evaluated in patients with albinism but studies on patients with low vision due to age-related macular degeneration and diabetes have shown a positive effect on health-related quality of life [26]. Others have found moderate improvements in health-related quality of life after physical rehabilitation of patients with physical disabilities or chronic disease. The latter study concluded that a patient-centered approach should be used that focuses on acceptance of the disability [27]. Nevertheless, it does seem reasonable to assume that being able to live an independent life without too many worries is deemed desirable by most and hence an important outcome after rehabilitation.

There are some limitations to the study. First, all participants had received some sort of optical rehabilitation prior to study participation. For most participants, glasses were old or their need for filters had changed since they last had a check-up but none of the participants were naïve to optical rehabilitation. Thus, the study does not reflect the benefits of rehabilitation in patients with albinism per se, but it represents the effect of updated rehabilitation in patients. Moreover, we wanted to evaluate the effect of optical rehabilitation tailored to the individual patient. Therefore, patients were offered a broad range of glasses, contact lenses, filters, loupes, magnifiers, and binoculars to choose from based on individual needs. Thus, our study cannot be used to differentiate if one type of optical rehabilitation results in greater improvement in vision-related quality of life than another type of optical rehabilitation for patients with albinism. Second, due to the limited number of translated and validated questionnaires for assessing the vision-related quality of life in patients with low vision in Danish, the VFQ39 questionnaire was the only available option, although adding other questionnaires may have added to the strengths of the study.

## 5. Conclusions

In conclusion, we found that vision-related quality of life assessed by the VFQ39 questionnaire was relatively low in Danish patients with albinism but that optical rehabilitation improved vision-related quality of life by a substantial degree for a number of important items. The main learning from the study was that an individual approach is required when addressing a complex visual impairment that involves both low vision and photosensitivity and that optical rehabilitation has effects beyond what it aims to address; most significantly, independence and mental health were improved in patients. However, our study addresses the short-term consequences of optical rehabilitation, and studies with longer follow-up and preferably also observations on the use of optical aids in everyday situations are required to learn more on the effects of rehabilitation in a lifelong perspective.

## Figures and Tables

**Table 1:** Baseline characteristics of included participants.

	*n* (%)
Males/females	43 (55)/35 (45)
*Visual impairment (ETDRS letters (logMAR)):*	
None (≥70 (0.3))	12 (15.4)
Mild (60–69 (0.5))	16 (20.5)
Moderate (35–59 (1.0))	44 (56.4)
Severe (20–34 (1.3))	3 (3.8)
Blindness (≤19 (1.32)	3 (3.8)
*Contrast sensitivity:*	
Above normal (>1.95)	5 (7.2)
Normal (1.95–1.80)	44 (63.8)
Moderately reduced (1.79–1.60)	12 (17.4)
Severely reduced (<1.60)	8 (11.6)

Visual impairment was defined using the WHO criteria (https://www.who.int/news-room/fact-sheets/detail/blindness-and-visual-impairment accessed on 5 July 2023). Contrast sensitivity was not available in 9 patients, normal values for contrast sensitivity were taken from Mäntyjärvi et al. [19].

**Table 2:** VFQ-39 subscale scores before and after an updated optical rehabilitation.

	Before		After		*p*-Value
VFQ39 Item	Median (Range)	% Ceiling	Median (Range)	% Ceiling	
General health	70.8 (6.5–100)	3.8%	76.0 (25.0–100)	8.0%	0.5
General vision	60.0 (17.5–100)	2.6%	60.0 (13.0–95.5)	0%	0.4
Ocular pain	75.0 (25.0–100)	1.3%	87.5 (37.5–100)	44.0%	<0.001 *
Near activities	65.8 (12.5–100)	6.4%	67.8 (8.3–100)	2.0%	0.07 *
Distance activities	62.5 (12.5–100)	1.3%	64.6 (8.3–100)	6.0%	0.08 *
Vision specific:					
Social functioning	75.0 (8.3–100)	20.5%	91.7 (8.3–100)	28.0%	0.007 *
Mental health	55.0 (0–70.0)	0%	75.0 (20.0–100)	2.0%	<0.001 *
Role difficulties	50.0 (12.5–75.0)	0%	68.8 (18.8–100)	4.0%	<0.001
Dependency	50.0 (12.5–75.0)	0%	87.5 (37.5–100)	30.0%	<0.001 *
Color vision	100 (25.0–100)	74.4%	100 (25.0–100)	76.0%	0.8 *
Peripheral vision	100 (25.0–100)	32.1%	100 (25.0–100)	36.0%	0.3 *
Composite score	62.5 (14.2–77.0)	0	76.5 (20.6–99.6)	0	<0.001

Before optical rehabilitation 78 patients filled out the questionnaire, and 50 returned the VFQ39 questionnaire after optical rehabilitation. The “Before” numbers are for all 78 patients and the “After” values are for the 50 who returned the questionnaires. Numbers are given as median (range). % ceiling indicates the percentage of participants who scored the item to the maximum value of 100. For the participants with paired observations (*n* = 50), *p*-values were calculated using paired *t*-tests except for * when Wilcoxon signed-rank sum test was used due to non-normal distribution.

## Data Availability

The data presented in this study are available on request from the corresponding author. The data are not publicly available due to ethical and data protection reasons.
